# Increasing best practice data sharing at *PLOS Pathogens*

**DOI:** 10.1371/journal.ppat.1010021

**Published:** 2021-11-11

**Authors:** Lauren Cadwallader, Kasturi Haldar, Michael H. Malim

**Affiliations:** 1 Public Library of Science, San Francisco, California, United States of America and Cambridge, United Kingdom; 2 Boler-Parseghian Center for Rare and Neglected Diseases, University of Notre Dame, Notre Dame, Indiana, United States of America; 3 Department of Infectious Diseases, King’s College London, London, United Kingdom

## Introduction

*PLOS Pathogens* is a trusted venue for impactful research that readers everywhere rely on to view, download, and share. The journal empowers authors to decide how they want to share their work by removing barriers, taking action on the feedback from our community, and providing transparency every step of the way. As with all PLOS journals, *PLOS Pathogens* strives to be a conduit for good open science practices. However, we know that there are barriers to the adoption of open science practices [1], and our approach to increasing adoption should be driven by evidence and community need.

## Current data sharing at *PLOS Pathogens*

Since 2014, all PLOS journals have mandated the sharing of research data, and *PLOS Pathogens* has seen a steady increase in the use of repositories from 16% of research articles with data depositing in a repository in 2014 to 29% in 2020. *PLOS Pathogens* authors share their data in a number of different places—discipline-specific repositories (e.g., Sequence Read Archive and Gene Expression Omnibus), generalist repositories (e.g., Dryad and Figshare), code repositories (e.g., Github), and within the Supporting information accompanying an article. In fact, in 2020, 75% of research articles published in *PLOS Pathogens* shared at least some of the data underlying their research in the Supporting information.

Sharing data via a repository—either discipline specific or generalist—has benefits for both authors and readers. Authors are able to demonstrate their compliance with the increasing number of funder and institutional policies that require data sharing [3,4]. The dataset can be cited independently of the article, which can be tracked using the assigned DOI, demonstrating the impact and value of the research to funders. And sharing data in a repository has been linked to a 25% increase in citations to the article [5]. Readers benefit as they are able to find data more easily as it is indexed in search engines and aggregators. Licensing information is clear so the potential for reuse is immediately obvious. Finally, repositories offer the possibility of machine-readable datasets, allowing for computational methods to access and reuse data.

Depositing in a repository is the ideal way to meet the requirements of the findable, accessible, interoperable, reusable (FAIR) principles for research data [2]. The data become a research output itself. Depositing it in a repository makes it findable, citable, and available for the long term (in accordance with the repository’s preservation guarantees). It is clear that sharing data via a repository has tangible benefits for the community whether they are authors or readers and that *PLOS Pathogens* could, and should, do more to promote sharing of data in this way.

## Integration

To enable more *PLOS Pathogens* researchers to share their data via a repository, we are experimenting with solutions that will make data deposition easier and more efficient. We have integrated the journal’s submission system with the Dryad repository (https://datadryad.org), which will be available for 1 year initially. Dryad is a generalist repository that will accept any data, with the exception of identifiable human data, as part of the manuscript submission process. Dryad will curate the dataset to ensure that it meets the minimum requirement, and *PLOS Pathogens* will cover the costs of this service for an unlimited number of authors for the initial 1-year period. These actions aim to further remove barriers to submission and therefore promote robust participation.

This integration is part of a Wellcome Trust–funded PLOS open science initiative to explore how PLOS can improve data sharing and engagement with the data that have been shared [6].

## Evidence-based solutions

This approach was chosen because research conducted by PLOS, involving mostly PLOS authors, suggests that busy researchers are unlikely to significantly alter their behavior when it comes to data sharing [7] and are more likely to participate in public data sharing if the barriers to entry (including time, multiple processes, and cost) are reduced or removed. Our view is that increased data sharing is more likely to occur if good practices are embedded into existing author workflows, such as the journal manuscript submission. By introducing a small, simple, and optional extra step to the submission process, we aim to promote good open science practice in a way that respects author choice and does not increase workload.

*PLOS Pathogens* is well placed to trial Dryad as the research data authors commonly put into Supporting information are primarily not suited to a discipline-specific repository but could be of value to other researchers who may wish to reproduce or utilize their results. Not all the outputs shared via Supporting information are suitable for a repository (e.g., tables supporting statistical analysis presented in the article), and so Supporting information will still be an option for authors wanting to share other materials.

## Success measures

The Dryad integration with *PLOS Pathogens* will initially be available for 1 year from October 2021 to all authors starting a new submission. During this time, we will be monitoring the uptake of the integration and conducting research with the community to further understand their attitudes toward data repositories and their experience of the integration. What we do after the first year will depend on what the community tell us during our consultations and if there has been a measurable increase in data sharing via a repository in the journal. We hope that this integration will empower *PLOS Pathogens* authors to practice data sharing in a way that benefits them and their community.
